# Systemic toxicity assessment of tissue conditioners modified with nystatin and chlorhexidine in a rat model of denture stomatitis

**DOI:** 10.1590/1678-7765-2025-0811

**Published:** 2026-05-22

**Authors:** Gustavo Simão Moraes, Thaís Albach, Carolina Yoshi Campos Sugio, Victoria Schlumberger Cachoeira, Falyne Kiratcz, Eduardo Bauml Campagnoli, Fabio André Dos Santos, Karin Hermana Neppelenbroek, Vanessa Migliorini Urban

**Affiliations:** 1 Universidade Estadual de Ponta Grossa Departamento de Odontologia Ponta Grossa Paraná Brasil Universidade Estadual de Ponta Grossa, Departamento de Odontologia, Ponta Grossa, Paraná, Brasil.; 2 Universidade de São Paulo Faculdade de Odontologia de Bauru Bauru São Paulo Brasil Universidade de São Paulo, Faculdade de Odontologia de Bauru, Bauru, São Paulo, Brasil.; 3 Universidade Federal de Uberlândia Faculdade de Odontologia Uberlândia Minas Gerais Brasil Universidade Federal de Uberlândia, Faculdade de Odontologia, Uberlândia, Minas Gerais, Brasil.

**Keywords:** Denture stomatitis, Dental tissue conditioning, Chlorhexidine, Nystatin, beta-Cyclodextrins

## Abstract

Denture stomatitis is a condition that commonly affects the palatal mucosa of denture wearers. Recently, several treatments have shown promising results, including the addition of antimicrobial drugs to tissue conditioners. However, before initiating clinical trials, the safety profile of this modified material must be assessed.

**Objective:**

This study investigated the potential systemic toxicity of a tissue conditioner modified with antifungals in a rat model of denture stomatitis.

**Methodology:**

Male Wistar rats (N=59) were randomly assigned to the following groups: NC (negative control), SD (sterile device), DS (denture stomatitis), Soft (Softone), Nys (nystatin), Nys:βCD (nystatin:β-cyclodextrin), Chx (chlorhexidine), and Chx:βCD (chlorhexidine:β-cyclodextrin). Animals of all groups, except for NC and SD, wore a palatal device contaminated with *Candida albicans* for the induction of denture stomatitis. Rats from the Soft group had their devices relined with a tissue conditioner (Softone) with no drugs added, while devices from groups Nys, Nys:βCD, Chx, and Chx:βCD were relined with antifungals at their minimum inhibitory concentrations against *C. albicans* SC5314 (32 mg, 36 mg, 64 mg, and 46 mg for each gram of tissue conditioner, respectively). Four days after treatment, the animals had their blood collected for biochemical analyses and had their livers, lungs, stomachs, and kidneys removed for histopathological and enzymatic analyses.

**Results:**

No significant renal, gastric, or pulmonary changes were detected. Some rats from groups Nys, Nys:βCD, Chx, and Chx:βCD presented vacuolation of hepatocytes, although this is not necessarily a sign of hepatic damage. However, the livers of the Chx group presented higher myeloperoxidase activity when compared to the NC, SD, and DS groups.

**Conclusion:**

These findings suggest that incorporating Nys, Nys:βCD, and Chx:βCD into this tissue conditioner may represent a safe option for denture stomatitis treatment.

## Graphical abstract

**Figure:**
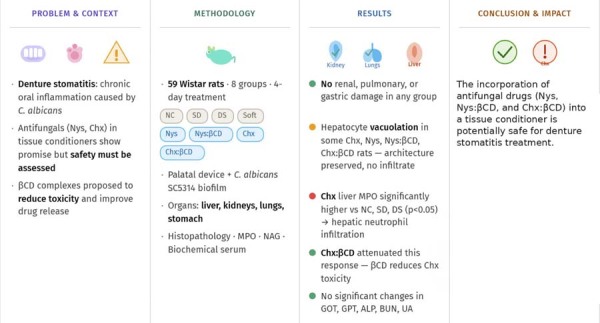

## INTRODUCTION

Denture stomatitis is a prevalent oral inflammatory condition marked by chronic, diffuse erythema and edema of the mucosa beneath dentures.^1,2^ Although its etiology is multifactorial, *Candida albicans* is recognized as the primary causative agent,^3^ largely due to its ability to form complex biofilms on denture base acrylic surfaces,^4^ which play a central role in treatment failure and the high incidence of relapse.^5^

Effective treatment of denture stomatitis requires sustained-release antifungal therapy that maintains adequate drug concentrations to reduce *Candida* colonization.^6^ In this regard, the incorporation of antifungal agents, including nystatin (Nys) and chlorhexidine (Chx), into denture relining materials has been suggested and has yielded promising results.^7-10^ This strategy relies solely on patients wearing their dentures, thereby minimizing the need for active patient compliance.^9,11^ Moreover, the reliner serves as a barrier between the contaminated inner denture surface and the oral tissues, thereby disrupting the cycle of reinfection.^12^

However, certain concentrations of Chx are associated with harmful effects, such as hepatotoxicity and nephrotoxicity,^13^ weight loss and dehydration,^14^ adrenergic nerve damage,^15^ respiratory complications, including pulmonary edema and hemorrhage,^16^ and DNA damage.^17^ Inadvertent administration of Chx in humans has caused severe reactions, including anaphylactic shock, dermatitis and stomatitis,^18^ pharyngeal edema, necrotic lesions in the esophagus and acute liver damage,^19^ gastritis,^20^ acute respiratory distress syndrome,^21^ and cardiac arrest.^22^ Nys, by contrast, is poorly absorbed from the gastrointestinal tract and is mostly excreted in the feces.^23^ Despite having few side effects, such as nausea,^24^ vomiting, and gastrointestinal discomfort,^25^ Nys can be toxic at concentrations above five million units, particularly toward nephrotic cells, which are vulnerable to the hemolytic action of polyene macrolides.^26,27^

In light of systemic toxicity concerns, cyclodextrin inclusion complexes have been proposed, offering the dual benefit of reducing drug toxicity and enhancing drug performance by increasing stability, overcoming solubility and bioavailability limitations, and enabling controlled antifungal release.^28^ Recent studies have demonstrated the effectiveness and biocompatibility of chlorhexidine:β-cyclodextrin and nystatin:β-cyclodextrin inclusion complexes both *in vitro*^8,10,29^ and *in vivo*.^30,31^These complexes improved antimicrobial activity by effectively inhibiting *Candida albicans* growth without inducing cytotoxic effects in fibroblasts.^29^ Moreover, their incorporation into a soft tissue conditioner significantly reduced the *Candida* load and decreased tissue inflammation in rats with denture stomatitis.^30,31^

Nevertheless, the *in vivo* safety of this modified soft tissue conditioner still needs to be assessed before clinical trials are initiated. In this context, the present study evaluated the potential systemic toxicity of a tissue conditioner modified with antifungal agents either complexed or not with β-cyclodextrin in a rat model of denture stomatitis.

## METHODOLOGY

### Ethical concerns

This study was carried out in accordance with the Guiding Principles for the Care and Use of Animals and the Animal Research: Reporting of *In Vivo* Experiments (ARRIVE) guidelines,^32^ and it received approval from the Ethics Committee on Animal Use of the State University of Ponta Grossa (UEPG, process number: 009/2019).

### Sample size

This study investigated the systemic implications of drug-modified tissue conditioners in the same animals used in a previous study that reported the effectiveness of these treatments.^30^This approach was adopted following the 3Rs principles in animal research: Replacement, Reduction, and Refinement, which aim to minimize or avoid animal use and improve welfare by reducing suffering, thus ensuring ethically conducted scientific outcomes.^33^

Wistar rats were randomly allocated to the experimental groups, as presented in Table 1. Sample size was calculated using G*Power 3.1 (Universität Düsseldorf, Düsseldorf, North Rhine-Westphalia, Germany). Because treatment effectiveness was the primary outcome, the estimation was based on *Candida albicans* colony-forming unit (CFU) counts obtained from oral samples of rats with experimentally induced denture stomatitis in a previous study.^34^ In that study, rats with denture stomatitis exhibited a mean *C. albicans* load of 12,850 CFU/mL. Assuming that the treatment groups (Nys, Chx, Nys:βCD, and Chx:βCD) would achieve a 90% reduction in oral *C. albicans* CFU counts, a sample size of five animals per group was estimated to provide a statistical power of 95% with an effect size of 2.31 (α=0.05). To account for potential losses, the number of animals per group was increased to seven.

**Table 1:** Main characteristics of experimental groups

Group	Acronym	Palatal device	Denture stomatitis	Tissue conditioner	Antifungal	Concentration*	n
Negative control	NC	-	-	-	-	-	4
Sterile device	SD	+	-	-	-	-	5
Denture stomatits	DS	+	+	-	-	-	3
Softone™	Soft	+	+	+	-	-	5
Nystatin	Nys	+	+	+	+	32 mg	5
Chlorhexidine	Chx	+	+	+	+	64 mg	4
Nystatin:ß-cyclodextrin	Nys:ßCD	+	+	+	+	36 mg	7
Chlorhexidine:ß-cyclodextrin	Chx;ßCD	+	+	+	+	46 mg	4

* Amount of antifungal drug per g of tissue conditioner. + (positive): present feature; - (negative): absent feature.

n: final number of animals per group after exclusions (3 lost during anesthetic procedures and 19 that lost their intraoral devices during the experimental period).

### Denture stomatitis model

The denture stomatitis model used in this study was previously described in another paper.^34^ Immunocompetent Wistar rats (N=59, 250–300 g, approximately 3 months old) were housed individually in plastic cages under controlled temperature (~22 °C) and a 12-h light/dark cycle. When necessary, general anesthesia was induced via intraperitoneal injection of 90 mg/kg of 10% ketamine (Ketalex; Rhobifarma Indústria Farmacêutica Ltda.) combined with 10 mg/kg of 2% xylazine (Xilazin; Syntec do Brasil Ltda.).

As an inclusion criterion, the animals had to be *Candida*-free.^35^ Starting seven days before infection, rats received 0.83 mg/mL of tetracycline hydrochloride (Amanda Manipulações Farmacêuticas) in their drinking water, available *ad libitum*.^34^

Acrylic resin devices were fabricated from impressions of the animal palates, which were obtained using individual trays and polyether. The devices were then contaminated with a *C. albicans* SC5314 suspension (~2.6 × 10⁷ CFU/mL) for 90 min, followed by incubation in RPMI 1640 medium at 37°C and 75 rpm for 48 h to allow biofilm formation.^36^ On the day of device placement, anesthesia was prolonged with an additional injection of 1% acepromazine maleate (Apromazin 1%; Syntec). For disease induction, rats were first orally infected with a suspension (~ 2 × 10^8^CFU/mL) of *C. albicans* using sterile swabs for 30 s. The contaminated acrylic resin devices were then fixed to their maxillary molars with a self-adhesive resin cement (RelyX U200; 3M ESPE) for four consecutive days.^36^

### Denture stomatitis treatment

The treatment protocol was also reported in another recent paper.^30^ After four consecutive days wearing the contaminated intraoral devices, the animals were anesthetized and the devices were removed. The internal surfaces of the devices were ground to a depth of 1 mm using a bur (KG Sorensen Ind. e Com. Ltda.) and subsequently relined following the procedures described by Hotta, et al.^37^ (2017).

The soft tissue conditioner (Softone; Bosworth Co.) was manipulated according to the manufacturer’s instructions, with or without the addition of the antifungal drugs. The antifungals used in this study were nystatin (Nys; Amanda Manipulações Farmacêuticas) and chlorhexidine (Chx; Sigma Aldrich). β-cyclodextrin (βCD; Sigma Aldrich) was used to prepare the inclusion complexes. Nystatin:β-cyclodextrin (Nys:βCD) and chlorhexidine:β-cyclodextrin (Chx:βCD) inclusion complexes were prepared at molar ratios of 1:1 and 1:2, respectively, in 50:50 (v/v) ethanol/distilled water, shaken at room temperature for 24 h, frozen at −82 °C, and lyophilized (LD1500A; Terroni) for 72 h.^10,38^

Antifungals were mixed with the powder material at concentrations that inhibit biofilm formation of *C. albicans* SC5314 (Nys: 32 mg; Nys:βCD: 36 mg; Chx: 64 mg; and Chx:βCD: 46 mg).^8,10^ Then, tissue conditioner liquid was added at the recommended ratio and the material was manipulated. After removal of excess material, the palatal devices were re-fixed to the maxillary molars. As controls, some animals did not wear intraoral devices (negative control – NC), used a sterile device (SD), used a contaminated device (DS – denture stomatitis), or used a device relined with the tissue conditioner without antifungals (Soft). Treatment lasted for four consecutive days, and animals that lost their devices were excluded from the study.

### Biochemical analyses

Before euthanasia, the animals were anesthetized and approximately 2 mL of blood was drawn by cardiac puncture and centrifuged at 5,000 rpm (5804 R; Eppendorf do Brasil Ltda.) for 7 min. The serum was then transferred to Eppendorf tubes and stored at −80°C. Biochemical parameters, including alkaline phosphatase (ALP), blood urea nitrogen (BUN), uric acid (UA), creatinine (CRE), glucose (GLU), glutamic oxalacetic transaminase (GOT), glutamic pyruvic transaminase (GPT), and amylase, were measured with a Random Access Analyzer (CT 600i; Wiener Lab Group).

### Histopathological analysis

After blood collection, rats were euthanized by cervical dislocation. The liver, stomach, kidneys, and lungs were carefully removed and divided into two parts.

A portion of each organ was fixed in 10% neutral buffered formalin (Dinâmica Química Contemporânea Ltda.) for histopathological analysis. After fixation for 24–48 h, tissues were routinely processed, paraffin-embedded, sectioned at 5 μm, and stained with hematoxylin and eosin. Slides were coded to ensure blinding, and histopathological evaluation was independently performed by two experienced examiners.

Overall, the occurrence of vasodilation and/or vasocongestion, and the presence of inflammatory exudate, hemorrhagic and/or necrotic areas, and signs of tissue inflammation were investigated. Each tissue was also assessed for specific alterations, including collapse of the alveoli in the lungs; vacuolation of the hepatocytes in the liver; disorganization of the epithelium and/or destruction of the gastric mucosa in the stomach; and collapsed tubules and altered glomeruli in the kidneys.

### Myeloperoxidase and N-acetylglucosaminidase assays

The remaining portion of each organ was stored in an ultra-freezer at −80°C. Samples were then prepared and analyzed following protocols described by Olchanheski, et al.^39^ (2018) and Ferreira, et al.^40^ (2007), with minor modifications as described below.

Myeloperoxidase (MPO) is an enzyme used as a marker of neutrophil infiltration into tissues. Tissue samples were homogenized in ice-cold 20 mM sodium phosphate buffer for 40 s and centrifuged at 13,000 rcf, 4 °C, for 10 min (5804 R; Eppendorf do Brasil Ltda.). The supernatants were discarded, and the resulting pellets were resuspended in 0.5% hexadecyltrimethylammonium bromide (HTAB) prepared in 50 mM sodium phosphate buffer. Following a second centrifugation under the same conditions, the supernatants were harvested for protein quantification by the Bradford assay. MPO activity was determined by measuring the hydrogen peroxide (H_2_O_2_)-dependent oxidation of 3,3′,5,5′-tetramethylbenzidine (Sigma-Aldrich). Absorbance was recorded every 2 min at 570 nm using a spectrophotometer (Ultrospec 1000a; Amersham Pharmacia Biotech Inc. – Molecular Dynamics Div.). MPO activity was expressed as optical density per milligram of protein.

N-acetylglucosaminidase (NAG) activity was assessed as an indicator of macrophage accumulation and/or activation.^41^ In a 96-well microplate, 100 μL aliquots of p-nitrophenyl-N-acetyl-D-glucosaminide (Sigma-Aldrich), prepared in Milli-Q water, were added to 100 μL of the remaining supernatant from each sample. The plates were incubated at 37 °C for 2 h, after which absorbance was measured at 405 nm using a spectrophotometer. NAG activity was expressed as optical density per milligram of protein.

### Statistical analysis

Normality and variance homogeneity were assessed with the Shapiro-Wilk and Levene tests, respectively. One-way ANOVA was applied to MPO values of the lungs and to BUN, GPT, GLU, and CRE values. Tukey’s HSD test followed ANOVA for GLU and CRE. Welch’s one-way ANOVA was used for MPO values of the stomach and liver, and for NAG values of the liver, lungs, and kidneys. When appropriate, the Games-Howell *post hoc* test followed Welch’s ANOVA. NAG values of the stomach and UA and GOT values were also analyzed using Welch’s ANOVA. All statistical tests were conducted at a 95% confidence level (α=0.05) using IBM SPSS Statistics for Windows, version 21.0 (IBM Corp., Armonk, NY, USA).

## RESULTS

### Animal welfare

Three animals (5.1%) were lost during anesthetic procedures, and nineteen (32.2%) lost their devices during the experiments and were therefore excluded from the analyses. No deaths were attributable to the treatment itself. Table 1 presents the final number of animals per group.

### Biochemical analyses

Table 2 presents the serum biochemical values after treatment. Significant differences were observed between GLU values of the NC and SD groups (p=0.032), and between CRE values of the SD and DS groups (p=0.013), DS and Soft (p<0.001), DS and Chx:βCD (p=0.014), and Soft and Nys:βCD groups (p=0.044).

**Table 2:** Biochemical parameters after the four-day treatment with the modified tissue conditioner

	NC	SD	DS	Soft	Nys	NysipCD	Chx	ChxipCD
**GLU (mmol/L)**	22.3 ±3.7^a^	13.7 ±4.2^b^	20.8 ±8.1^ab^	21.7 ±13.7^ab^	23.6 ±7.3^ab^	15.5 ±3.8^ab^	18.0 ±7.0^ab^	14.4 ±6.0^ab^
**BUN (mmol/L)**	10.4 ±3.4	8.7 ±1.2	9.9 ±0.3	9.7 ±1.6	8.8 ±1.6	8.4 ±0.9	9.8 ±3.0	8.0 ±1.4
**CRE (Mmol/L)**	33.4 ±4.9^abcde^	29.4 ±5.5^abc^	46.6 ±10.9^d^	23.4 ±6.2^ae^	33.2 ±7.3^abcde^	38.5 ±9.5^bd^	37.0 ±10.0^abcde^	29.4 ±7.1^ce^
**UA (Mmol/L)**	49.1 ±14.9	171.3 ±125.9	190.3 ±131.5	40.2 ±5.7	80.9 ±36.3	66.9 ±29.7	105.4 ±49.3	69.4 ±46.4
**GOT (IU/L)**	330.3 ±115.6	124.0 ±34.2	290.7 ±161.0	218.0 ±61.1	262.2 ±113.9	156.8 ±15.0	138.4 ±33.6	225.8 ±136.9
**GPT (IU/L)**	88.0 ±23.2	77.8 ±17.9	85.3 ±41.2	83.6 ±15.2	74.8 ±26.1	56.5 ±10.3	58.1 ±17.4	58.0 ±20.8
**ALP (IU/L)**	389.5 ±101.6	310.8 ±130.5	291.3 ±188.9	281.8 ±172.8	276.0 ±51.1	275.0 ±77.5	282.0 ±134.4	245.3 ±124.3
**Amylase (IU/L)**	895.5 ±165.2	605.0 ±64.7	647.7 ±203.4	550.6 ±226.3	631.6 ±153.5	625.3 ±90.5	550.4 ±257.1	588.3 ±374.4

Data represent the mean ±standard deviation. Means with different letters are significantly different at α=0.05.

GLU: glucose; BUN: blood urea nitrogen; CRE: creatinine; UA: uric acid; GOT: glutamic oxalacetic transaminase; GPT: glutamic pyruvic transaminase; ALP: alkaline phosphatase.

NC: negative control; SD: sterile device; DS: denture stomatitis; Soft: Softone; Nys: nystatin; NysßCD: nystatin:ß-cyclodextrin; Chx: chlorhexidine; Chx:ßCD: chlorhexidine:ß-cyclodextrin.

### Histopathological analysis

**Figure 1:**
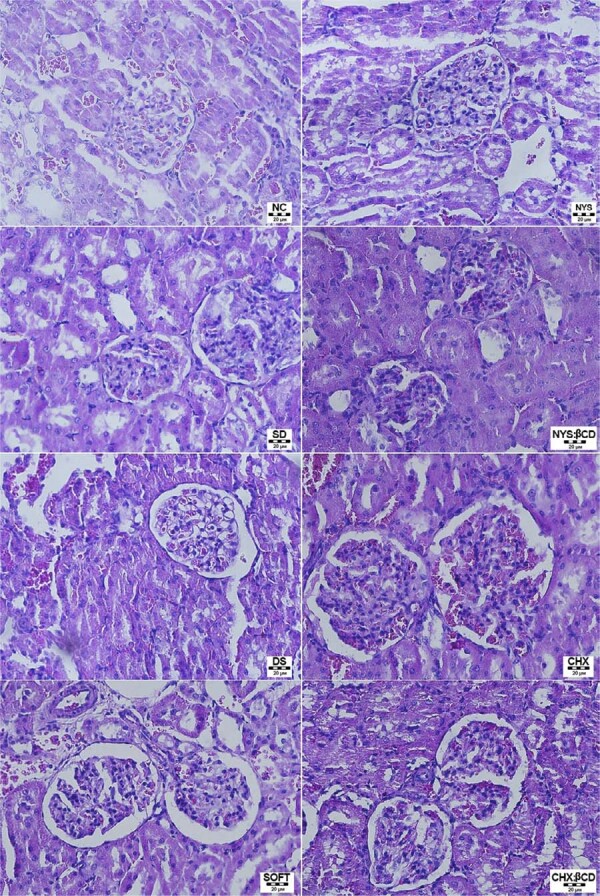
Histological images of the kidneys after the four-day treatment with the modified tissue conditioner. No noticeable changes were detected in the glomeruli. Hematoxylin and eosin stain – 400×

NC: negative control; SD: sterile device; DS: denture stomatitis; Soft: Softone; Nys: nystatin; Nys:βCD: nystatin:β-cyclodextrin; Chx: chlorhexidine; Chx:βCD: chlorhexidine:β-cyclodextri

**Figure 2:**
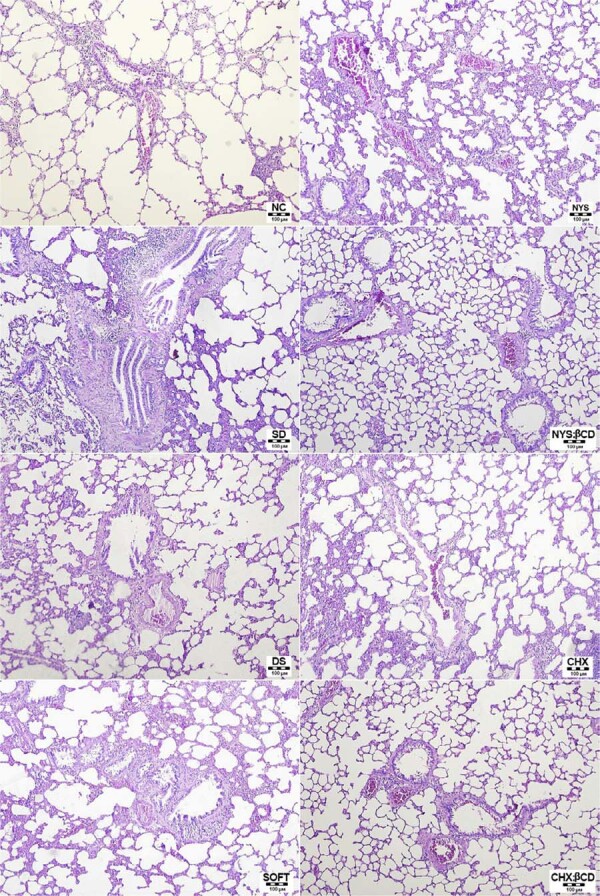
Histological images of the lungs after the four-day treatment with the modified tissue conditioner. No noticeable changes were detected in the alveoli. Hematoxylin and eosin stain – 200×

NC: negative control; SD: sterile device; DS: denture stomatitis; Soft: Softone; Nys: nystatin; Nys:βCD: nystatin:β-cyclodextrin; Chx: chlorhexidine; Chx:βCD: chlorhexidine:β-cyclodextrin.

**Figure 3:**
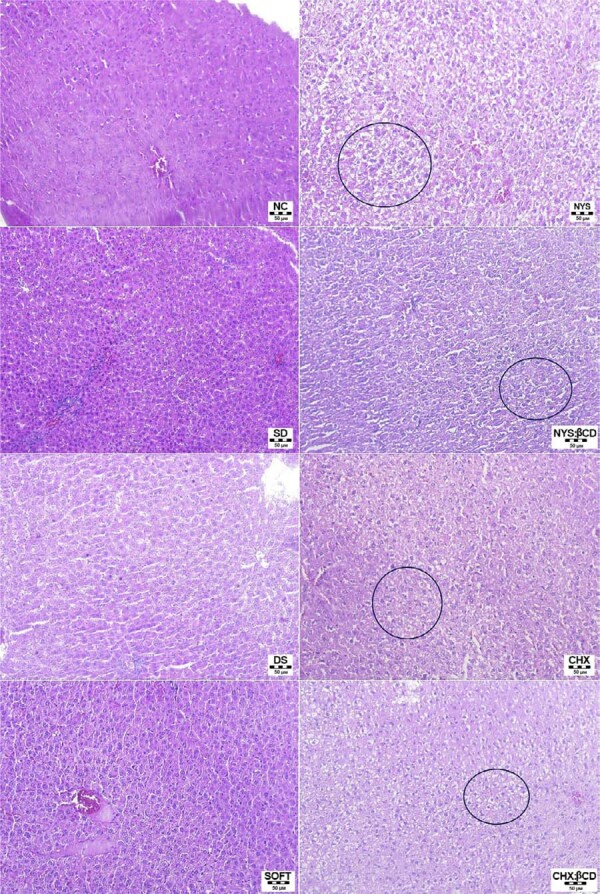
Histological images of the livers after the four-day treatment with the modified tissue conditioner. Black circles indicate areas of vacuolation of hepatocytes. Hematoxylin and eosin stain – 200×

NC: negative control; SD: sterile device; DS: denture stomatitis; Soft: Softone; Nys: nystatin; Nys:βCD: nystatin:β-cyclodextrin; Chx: chlorhexidine; Chx:βCD: chlorhexidine:β-cyclodextrin.

No apparent alterations were observed in the kidneys (Figure 1) or lungs (Figure 2). However, six rats (three from the Chx, one from the Nys, one from the Nys:βCD, and one from the Chx:βCD groups) presented vacuolation of hepatocytes, although tissue architecture was preserved and there were no signs of inflammatory infiltrate (Figure 3).

**Figure 4:**
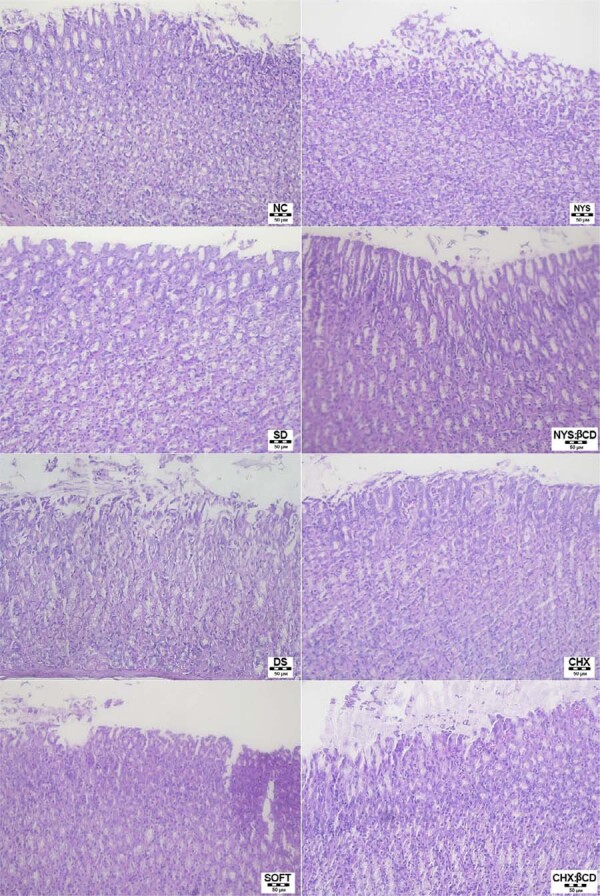
Histological images of the stomach mucosa after the four-day treatment with the modified tissue conditioner. Hematoxylin and eosin stain – 200×

NC: negative control; SD: sterile device; DS: denture stomatitis; Soft: Softone; Nys: nystatin; Nys:βCD: nystatin:β-cyclodextrin; Chx: chlorhexidine; Chx:βCD: chlorhexidine:β-cyclodextrin.

Some rats from the DS (n=1), Chx (n=2), and Chx:βCD (n=2) groups showed the presence of cells very similar to *Candida* yeast in the final portion of the esophagus, but no noticeable changes were observed in the gastric epithelium or mucosa of any animal (Figure 4).

### Myeloperoxidase and N-acetylglucosaminidase assays

**Figure 5:**
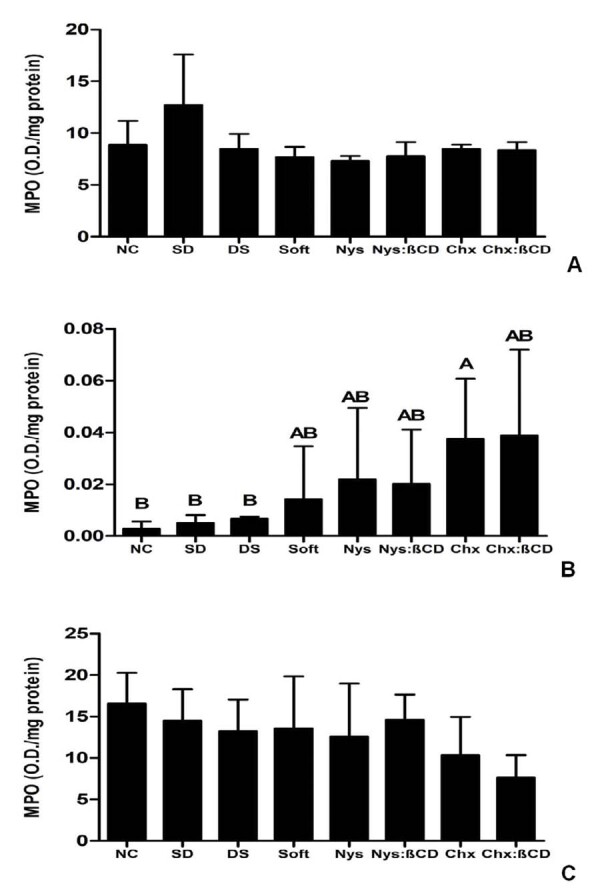
Myeloperoxidase (MPO) activity after treatment. Bars represent mean values, and error bars indicate standard deviations. Means with different capital letters are significantly different (α=0.05)

No significant differences were found in MPO activity of the stomach or lungs (p>0.05, Figure 5A, B). The livers of rats from the Chx group presented significantly higher MPO activity compared to the NC (p=0.009), SD (p=0.014), and DS (p=0.028) groups (Fig. 5C). It was not possible to measure MPO activity in the kidneys.

A: stomach; B: lungs; C: liver . NC: negative control; SD: sterile device; DS: denture stomatitis; Soft: Softone; Nys: nystatin; Nys:βCD: nystatin:β-cyclodextrin; Chx: chlorhexidine; Chx:βCD: chlorhexidine:β-cyclodextrin.

**Figure 6:**
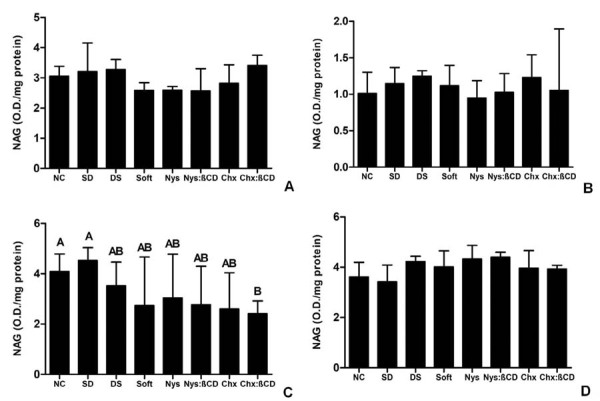
N-acetylglucosaminidase (NAG) activity following treatment. Bars indicate mean values, and error bars represent standard deviations. Means labeled with different capital letters are significantly different (α=0.05)

No significant differences in NAG activity were observed in the stomach or liver (p>0.05, Figure 6A, B). The lungs of the Chx:βCD group presented significantly lower NAG activity compared to the NC (p=0.035) and SD (p=0.002) groups (Figure 6C). A significant difference was observed between NAG activity in the kidneys of the Chx:βCD and Nys:βCD groups (p=0.033) (Figure 6D).

A: stomach; B: liver; C: lungs; D: kidneys. NC: negative control; SD: sterile device; DS: denture stomatitis; Soft: Softone; Nys: nystatin; Nys:βCD: nystatin:β-cyclodextrin; Chx: chlorhexidine; Chx:βCD: chlorhexidine:β-cyclodextrin.

## DISCUSSION

The presence of hepatocyte vacuolation suggests that the drug-modified tissue conditioners may induce mild, initial hepatic alterations in some animals from the Chx, Nys, Chx:βCD, and Nys:βCD groups. However, preservation of overall tissue architecture, absence of inflammatory infiltrate, and lack of changes in biochemical parameters (GOT, GPT, and ALP—commonly used as indicators of liver injury^13^) indicate that this damage was not severe. Although chlorhexidine has been reported to significantly increase GOT and GPT levels in both animals^13^ and humans,^19^ elevated enzyme levels were not observed in the present study. Moreover, hepatocyte vacuolation, which is frequently associated with mild acute or subacute liver injury, may represent an adaptive cellular response rather than a degenerative process.^42^

Notably, the Chx group also exhibited significantly higher MPO activity than the NC, SD, and DS groups, suggesting a greater degree of hepatic inflammation. In contrast, this response was attenuated in the Chx:βCD group, reinforcing previous evidence that cyclodextrin inclusion complexes can reduce drug-related toxicity. This difference may also be related to the release profile of chlorhexidine from the tissue conditioner. By comparison, nystatin is generally regarded as safe and unlikely to induce hepatic injury.^43^ Finally, it should be considered that the hepatic alterations observed may reflect a combined or synergistic effect of anesthetic agents, tetracycline, and the antifungal drugs incorporated into the tissue conditioner.

The modified tissue conditioners did not cause any microscopic or biochemical (BUN, UA, and CRE) abnormalities in the kidneys, apart from higher CRE values in the Nys:βCD group compared to the Soft group. It was not possible to detect renal MPO activity, likely due to the extraction method employed. Kidney tissue has been reported to lack detectable MPO activity unless modifications to the original technique are performed.^44^ Previous studies have identified the kidney as a target organ for Chx toxicity.^17,45^ Xue, et al.^45^ (2011) reported a significant increase in CRE levels seven days after rats were exposed to 0.2% Chx,^16^ while Chow, et al.^13^ (1977) noted elevated BUN activity 24 h after administration of 1 g/kg of Chx. Nys has been reported to induce elevated BUN and CRE levels in mice following administration of a 5 mg/kg dose for three consecutive days.^46^

According to Conti, et al.^47^ (2015), the stomach, esophagus, and intestines are the primary sites of *Candida* colonization in mice. Despite the presence of some yeast-like cells consistent with *Candida* in the final portion of the esophagus (data not shown), no apparent changes were detected in the gastric epithelium or mucosa. Chx can be extremely harmful to both the esophagus and stomach at high concentrations, causing severe necrosis and congestion.^19,45^ A case report described vomiting, multiple gastric erosions, and acute gastritis after the ingestion of 4% chlorhexidine gluconate.^20^ A key point to consider is that these studies reported ingestion of Chx at much higher concentrations, which may explain why no animals developed signs of stomach damage in the present study. Nys is poorly absorbed from the gut and only causes occasional gastrointestinal symptoms, such as nausea, at high therapeutic doses.^48^

The lungs showed no signs of damage, either in histopathological analysis or in the enzymatic (MPO and NAG) assays, probably due to the low drug concentrations included in the tissue conditioner. At various concentrations, Chx administration can cause dyspnea, cessation of breathing, congestion of the alveoli,^45^ intra-alveolar edema, hemorrhages,^16^ and acute respiratory distress syndrome.^21^ There are no reports of lung damage caused by Nys. In fact, Nys can be successfully used for the prevention^49^ and treatment^50^ of fungal pulmonary infections.

It is important to acknowledge the limitations intrinsic to animal studies, particularly those related to metabolic differences between rats and humans. These limitations include the short treatment duration, which was limited to four days due to the animals’ inherent ability to eliminate *C. albicans*, potentially introducing interpretation bias. Animal losses during the experimental period and the inability to detect renal myeloperoxidase (MPO) activity should also be considered as limitations.

## CONCLUSIONS

Our findings indicate that incorporating antifungal agents, particularly Nys, Nys:βCD, and Chx:βCD, into the evaluated tissue conditioner represents a potentially safe therapeutic strategy for the management of denture stomatitis.
